# Combinational Spinal GAD65 Gene Delivery and Systemic GABA-Mimetic Treatment for Modulation of Spasticity

**DOI:** 10.1371/journal.pone.0030561

**Published:** 2012-01-23

**Authors:** Osamu Kakinohana, Michael P. Hefferan, Atsushi Miyanohara, Tetsuya Nejime, Silvia Marsala, Stefan Juhas, Jana Juhasova, Jan Motlik, Karolina Kucharova, Jan Strnadel, Oleksandr Platoshyn, Peter Lazar, Jan Galik, Laurent Vinay, Martin Marsala

**Affiliations:** 1 Neuroregeneration Laboratory, Department of Anesthesiology, University of California San Diego, La Jolla, California, United States of America; 2 Gene Therapy Program and Department of Pediatrics, University of California San Diego, La Jolla, California, United States of America; 3 Institute of Animal Physiology and Genetics, AS CR, Liběchov, Czech Republic; 4 Sanford-Burnham Medical Research Institute, La Jolla, California, United States of America; 5 Department of Breeding and Diseases of Game and Fish, University of Veterinary Medicine and Pharmacy, Komenskeho, Košice, Slovakia; 6 Institute of Neurobiology, Slovak Academy of Sciences, Košice, Slovakia; 7 Faculty of Science, Institute of Biology and Ecology, Pavol Jozef Safarik University, Košice, Slovakia; 8 Laboratoire Plasticité et Physio-Pathologie de la Motricité (UMR6196), Centre National de la Recherche Scientifique (CNRS) and Aix-Marseille Université, Marseille, France; Emory University, United States of America

## Abstract

**Background:**

Loss of GABA-mediated pre-synaptic inhibition after spinal injury plays a key role in the progressive increase in spinal reflexes and the appearance of spasticity. Clinical studies show that the use of baclofen (GABA_B_ receptor agonist), while effective in modulating spasticity is associated with major side effects such as general sedation and progressive tolerance development. The goal of the present study was to assess if a combined therapy composed of spinal segment-specific upregulation of GAD65 (glutamate decarboxylase) gene once combined with systemic treatment with tiagabine (GABA uptake inhibitor) will lead to an antispasticity effect and whether such an effect will only be present in GAD65 gene over-expressing spinal segments.

**Methods/Principal Findings:**

Adult Sprague-Dawley (SD) rats were exposed to transient spinal ischemia (10 min) to induce muscle spasticity. Animals then received lumbar injection of HIV1-CMV-GAD65 lentivirus (LVs) targeting ventral α-motoneuronal pools. At 2–3 weeks after lentivirus delivery animals were treated systemically with tiagabine (4, 10, 20 or 40 mg/kg or vehicle) and the degree of spasticity response measured. In a separate experiment the expression of GAD65 gene after spinal parenchymal delivery of GAD65-lentivirus in naive minipigs was studied. Spastic SD rats receiving spinal injections of the GAD65 gene and treated with systemic tiagabine showed potent and tiagabine-dose-dependent alleviation of spasticity. Neither treatment alone (i.e., GAD65-LVs injection only or tiagabine treatment only) had any significant antispasticity effect nor had any detectable side effect. Measured antispasticity effect correlated with increase in spinal parenchymal GABA synthesis and was restricted to spinal segments overexpressing GAD65 gene.

**Conclusions/Significance:**

These data show that treatment with orally bioavailable GABA-mimetic drugs if combined with spinal-segment-specific GAD65 gene overexpression can represent a novel and highly effective anti-spasticity treatment which is associated with minimal side effects and is restricted to GAD65-gene over-expressing spinal segments.

## Introduction

Spinal cord injury (traumatic or ischemic) may lead to the development of clinically-defined spasticity and rigidity [1], [2]. One of the underlying mechanisms leading to the appearance of spasticity after spinal injury is believed to be the loss of local segmental inhibition and the resulting: i) increase in tonic motoneuron firing [3], [4], ii) increase in primary afferent input during muscle stretch [5], and/or iii) exacerbated responses to peripheral sensory stimulation (i.e., allodynia) [6], [7]. Loss of GABA-mediated presynaptic, recurrent and reciprocal postsynaptic inhibition [3] as well as the loss of its inhibitory effect in flexor afferent pathways has been shown to represent one of the key mechanisms [8], [9], [10], [11].

Interestingly, however, previous studies have shown a significant increase in spinal parenchymal GAD67 expression in lumbar spinal segments in Th12 transected cats [12]. Similarly, an increased density of inhibitory boutons apposing α-motoneuron membranes has been shown in adult rats with midthoracic spinal cord transection performed at postnatal day 5 [13]. These data suggest that a static increase in GABA synthesizing enzymes in spinal interneurons or increase in the number of inhibitory contacts with α-motoneurons after spinal trauma, in the absence of a specific inhibitory neuron-driven activity, is not sufficient to prevent the development of spasticity/hypereflexia. In addition to the role of decreased inhibition, several other potential mechanisms have been shown to contribute to the development of spasticity after spinal trauma, including: **i)** progressive increase in α-motoneuronal 5-HT_2C_ receptor activity which became spontaneously active in the absence of brain-derived serotonin [14], or **ii)** the down regulation of the potassium-chloride co-transporter KCC2 in motoneurons and resulting switch to GABA-mediated depolarization [3]. Jointly, these data indicate that the mechanism leading to the development of spasticity after spinal injury (traumatic or ischemic) is complex and can vary depending on the model used as well as the age of experimental animals when the injury is induced.

Clinical pharmacological-treatment studies show that the use of systemic or spinally-administered baclofen (GABA_B_ receptor agonist) represents the most potent anti-spasticity pharmacological treatment. While effective in modulating spasticity of different etiologies including spinal trauma, amyotrophic lateral sclerosis or central stroke, major side effects such as general sedation and progressive tolerance development often limit its chronic use [15], [16], [17]. The use of systemically-administered GABA-mimetic compounds such as tiagabine (GABA reuptake inhibitor) shows only a weak or no anti-spasticity effect in clinically-acceptable doses [18], which correlates with a relatively modest potentiation of brain [19], [20] or spinal parenchymal GABA release after systemic delivery (current data). In addition, currently available spinal drug delivery systems (such as epidural or intrathecal delivery) do not permit a spinal segment-restricted therapeutic effect. Because the origin of spasticity affecting individual muscle groups can be somatotopically mapped to specific spinal segments, the development of segment-targeted anti-spasticity treatments would represent a clear advantage over current therapeutic approaches by reducing unwanted side effects.

We therefore analyzed: i) if a combined treatment composed of spinal segment-specific upregulation of GAD65 (glutamate-decarboxylase) gene and systemic delivery of tiagabine (GABA uptake inhibitor) in rats with ischemia-induced spasticity will lead to an antispasticity effect, and ii) whether such a combined treatment will be specific for GAD65 gene overexpressing spinal segments.

## Results

### Loss of GABA-ergic interneurons and upregulation of α-motoneuronal GABA B R1 and R2 receptor in animals with ischemic spasticity

**Figure 1:**
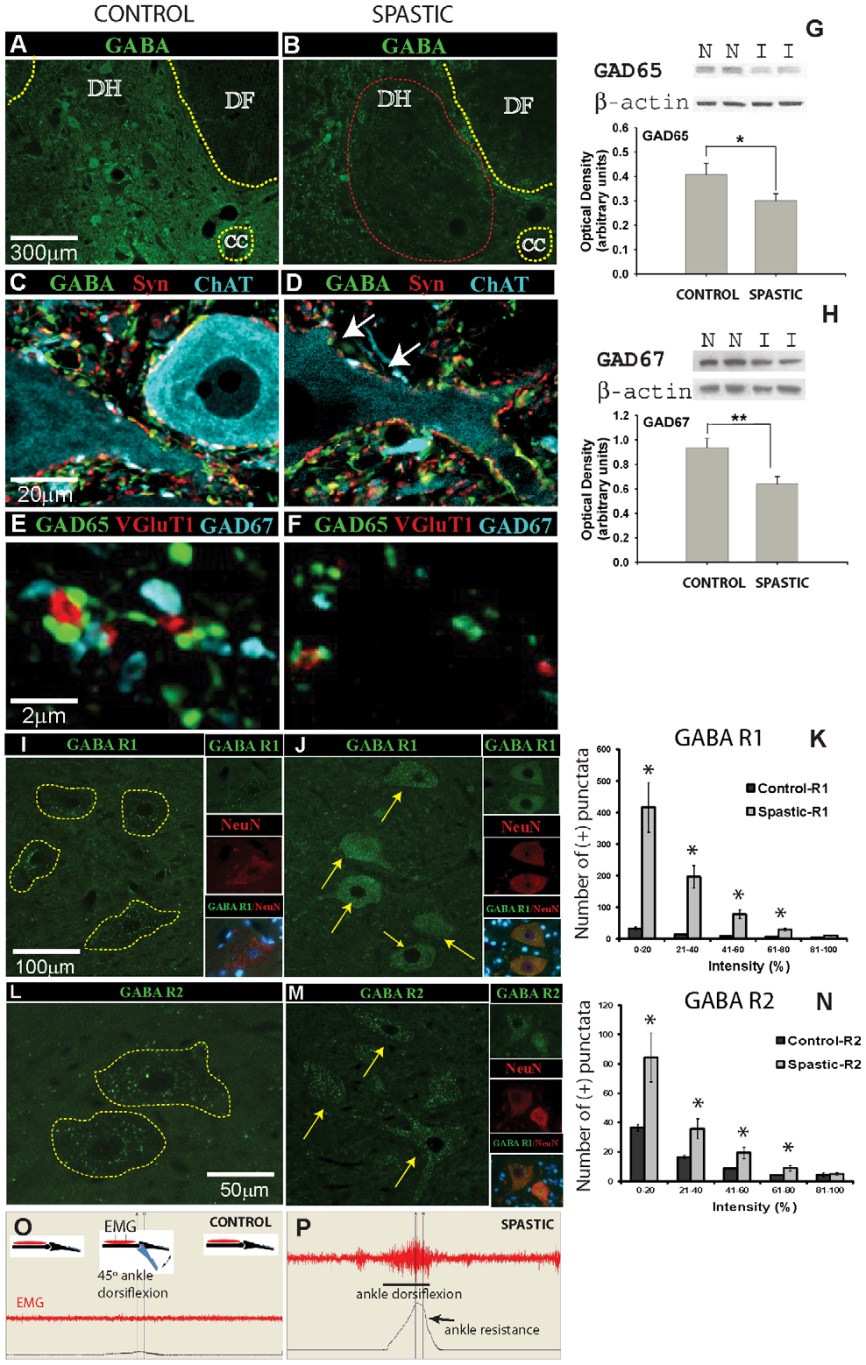
Loss of segmental inhibitory GABA-ergic interneurons and increased expression of GABA B R1+R2 receptor in α-motoneurons after transient spinal cord ischemia is associated with the development of chronic muscle spasticity. (**A, B**) Transverse spinal cord sections taken from L2–L5 segments in control (**A**) or spinal ischemia-induced-spastic rat (**B**) at 24 h after intrathecal colchicine injection and stained for GABA. Note an apparent loss of GABA-ergic interneurons in the intermediate zone in spastic rat (**B**; red circle). (**C–F**) Loss of GABA-ergic interneurons corresponds with loss of GABA-IR and GAD65-IR boutons on membranes of persisting CHAT-IR α-motoneurons in animals with ischemic spasticity (white arrows). (**G, H**) Western blotting for GAD65 and GAD67 in lumbar spinal cord samples taken from control animals (n = 5–6) or animals with developed ischemic spasticity (n = 5–6), (*P = 0.017; **P = 0.045, unpaired *t*-test). (**I–N**) In comparison to control animals, an upregulation in GABA B R1+R2 receptors in lumbar α-motoneurons was identified in animals with spasticity (compare **Fig. 1I** to **Fig. 1J** and **Fig. 1L** to **Fig. 1M**). Quantitative densitometric analysis showed significantly increased densities for both receptor subunits in spastic animals (*-P<0.05; **Fig. 1K** and **Fig. 1N**). (**O, P**) Measurement of EMG activity in gastrocnemius muscle and corresponding ankle resistance during computer-controlled ankle rotation (45°/3 sec) in awake control sham-operated animals (**O**) and in animals with ischemic spasticity (**P**).

We first quantified the loss of GABA-ergic neurons and bouton-like terminals in laminae VII and IX of lumbar spinal cord sections taken from spastic and sham-operated control animals. In comparison with control animals spastic animals had 50% less GABA-ergic neurons (average 39.0±13.4 vs. 19.5±4.2 per section; p<0.01; **Fig. 1A, B**) in lamina VII. In those same sections, GABA-ergic contact with α-motoneurons was also assessed, revealing a significant reduction in spastic animals (**Fig. 1D**-white arrows): 19.4±0.9 GABA/Syn-immunoreactive (IR) boutons contacting each motoneuron soma in control tissue compared to 10.1±0.8 in animals with spasticity (p<0.01; **Fig. 1C, D**). We next examined the number of GAD65 or GAD67 bouton-like structures that contacted VGluT1-IR primary afferent terminals in lamina IX (i.e. the site of GABAergic presynaptic Ia afferent inhibition). In control animals 26.7±4.2% of primary afferent terminals had clear GAD65 contact, compared to only 5.3%±1.2% in ischemia-injured tissue (p<0.001; **Fig. 1E, F**). No statistical difference was noted in the number of VGluT1 terminals that had GAD67 contact (25.3±4.8% in control vs. 15.2±4.3% in ischemic-spastic). No overall change in the number of VGluT1-IR terminals was noted in lamina IX (40.5±8.2 for control vs. 41.7±6.9 for ischemic-spastic). In the subgroup of VGluT1-IR structures that had GAD65 contacts (that is ignoring those with zero GAD65 contact), an average of 3.9±0.1 GAD65-IR boutons contacted each VGluT1 terminal in control sections. In spastic animals this number was reduced to 2.6±0.3 (p = 0.003). Similar analysis of GAD67 contact with VGluT1 terminals showed a trend towards reduction in animals with spasticity (1.8±0.1 in control vs. 1.2±0.06 in ischemic-spastic; p = 0.08). Western blotting of whole tissue homogenates from the lumbar spinal cord showed that, compared to control tissue, GAD65 protein was reduced by 26±5% (p = 0.045), and GAD67 by 32±6% (p = 0.017; **Fig. 1G, H**) in ischemic-spastic animals. We next analyzed the presence of GABA B R1 and R2 receptors on lumbar α-motoneurons using immunofluorescence staining in control animals and animals at 3 months after induction of ischemic spasticity (n = 3). In control animals a sparse punctate-like GABA B R1 and R2 immunoreactivity was identified in α-motoneurons. In contrast, animals with spasticity had a clear increase in GABA B R1 and R2 immunoreactivity on α-motoneuron membrane and in cytoplasm (**Fig. 1 I, J, L, M**). Densitometric image analysis showed a significant increase for both GABA B R1 and R2 receptor punctata in animals with spasticity if compared to controls (p<0.05; **Fig. 1K, N**).

Animals with spinal ischemic injury showed progressive development of extensor-type paraplegia with components of muscle spasticity and rigidity. To assess the presence of spasticity, ankle resistance and EMG activity in gastrocnemius muscle was measured during computer-controlled ankle dorsiflexion. Compared to control animals, a clear increase in muscle resistance and EMG activity in spastic animals was measured at 7–60 days after ischemic injury (**Fig. 1O, P**). In previous studies we have demonstrated a potent anti-spasticity effect after intrathecal baclofen delivery [21]. Jointly these data demonstrate that a selective loss of GABA-ergic segmental inhibition is the primary mechanism leading to the appearance of muscle spasticity and rigidity resulting from spinal ischemic injury. Accordingly, we hypothesized that an increase in the local spinal GABA-ergic tone (as achieved by spinal GAD65 gene delivery or intrathecal GABA delivery) in previously injured spinal segments should improve local inhibition and lead to amelioration of spasticity.

### Release of biologically active GABA from GAD65 over-expressing rat spinal cord primary cells or human fetal spinal cord astrocytes

**Figure 2:**
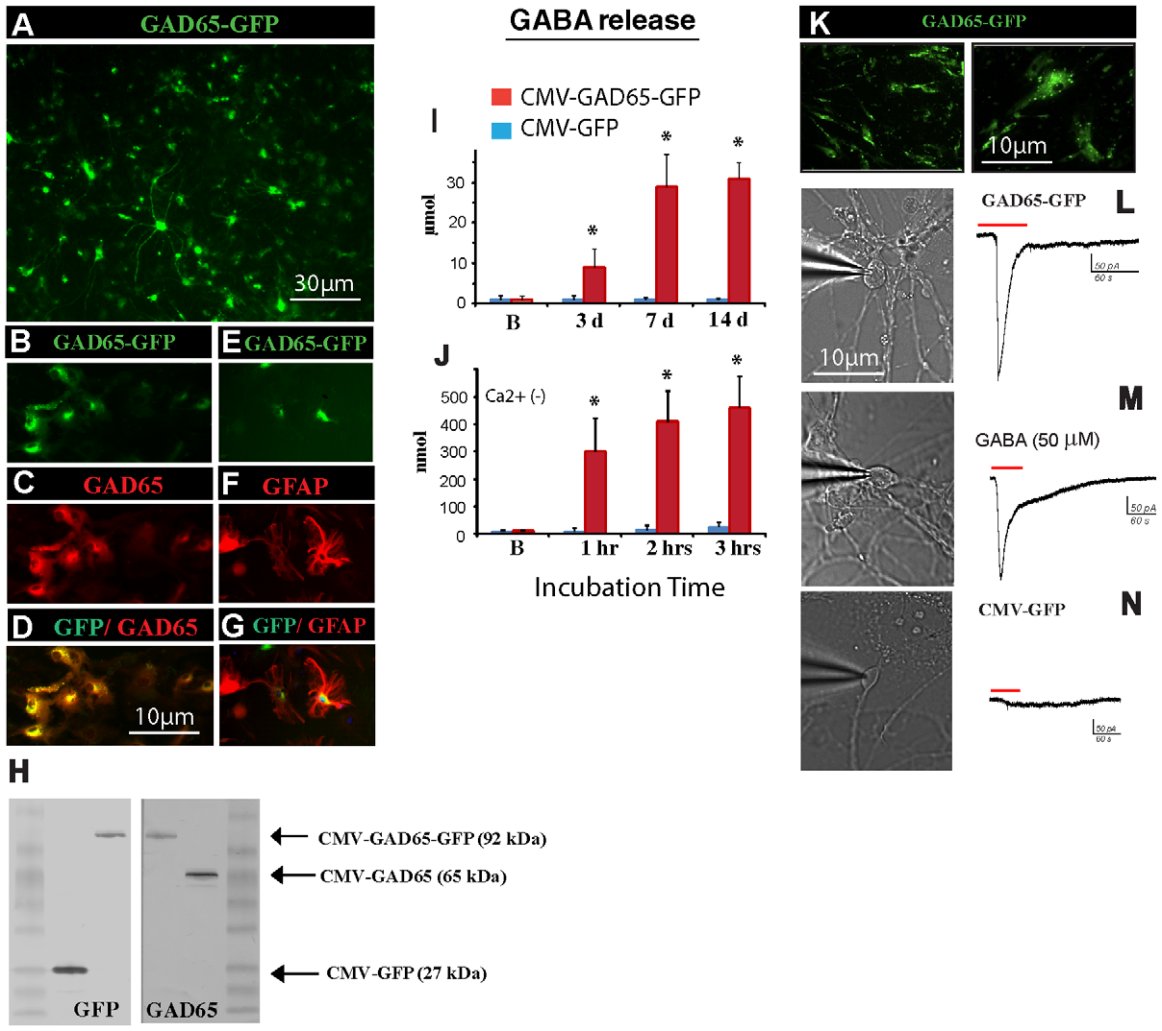
Infection of rat primary spinal cord culture with HIV1-CMV-GAD65 or HIV1-CMV-GAD65-GFP lentivirus leads to a preferential astrocyte GAD65 expression and release of biologically active GABA. (**A**) Rat spinal cord primary culture infected with HIV1-CMV-GAD65-GFP lentivirus and stained with anti-GFP antibody at 4 days after lentivirus infection. (**B–D**) Co-staining of HIV1-CMV-GAD65-GFP-infected cells with GAD65 antibody showed preferential GAD65 expression in GFP-IR cells. (**E–G**) Colocalization of GFP-IR with GFAP-IR in HIV1-CMV-GAD65-GFP-infected astrocytes at 14 days after infection. (**H**) Western blotting for GFP or GAD65 in cell lysates taken from rat primary spinal cord culture infected with HIV1-CMV-GFP (control), HIV1-CMV-GAD65-GFP, and HIV1-CMV-GAD65 lentivirus. (**I**) Extracellular GABA release measured in cell culture media taken from rat primary spinal cord culture 3–14 days after HIV1-CMV-GFP (control) or HIV1-CMV-GAD65-GFP lentivirus injection. (**J**) Progressive increase in extracellular GABA release measured in Ca^2+^-free media 1–3 hrs after cell culture wash in HIV1-CMV-GAD65-GFP but not in HIV1-CMV-GFP (control) lentivirus-infected cells (* P<0.01; paired *t* test). (**K**) Human fetal spinal cord astrocytes infected with HIV1-CMV-GAD65-GFP lentivirus and stained with anti-GFP antibody at 7 days after lentivirus infection. (**L**) Changes in whole-cell inward current in cultured human NT neurons after bath application of human astrocyte-HIV1-CMV-GAD65-GFP-conditioned media, (**M**) 50 µM GABA or (**N**) human astrocyte-HIV1-CMV-GFP-conditioned media (control); (neurons clamped at holding potential (-) 60 mV).

We next prepared and tested the efficacy of lentivirus-mediated GAD65 overexpression in primary rat spinal cord culture and resulting increase in extracellular GABA release. Cells were infected with HIV1-CMV-GAD65, HIV1-CMV-GAD65-GFP or HIV1-CMV-GFP lentivirus (LVs). At intervals longer than 3–4 days, clear GFP expression was seen in cells infected with GFP-tagged LVs (**Fig. 2A**). In HIV1-CMV-GAD65-GFP–infected cells, GFP expression showed colocalization with GAD65 (**Fig. 2B–D**) and was primarily expressed in astrocytes (**Fig. 2E–G**). Western blot analyses of cell cultures infected with each of the constructs confirmed the presence of GFP, GAD65 or the GAD65-GFP-fused protein (**Fig. 2H**). Media GABA concentrations from cell cultures infected with HIV1-CMV-GAD65-GFP (but not in HIV1-CMV-GFP-infected) showed a progressive and significant increase between 3–14 days after LV infection (baseline:150±65 nmol→27 µmol±9 at 7 days; p<0.01), (**Fig. 2I**). Replacing the culture media at 14 days after infection with Ca^2+^-free buffer showed Ca^2+^-independent increase in GABA release and was seen in HIV1-CMV-GAD65-GFP (baseline 4±3nmol→445±95 nmol at 3 hrs; p<0.01) but not in HIV1-CMV-GFP-infected cells (**Fig. 2J**).

We next tested if the GABA released from infected human fetal astrocytes is biologically active by measuring changes in inward current in patch-clamped human NT neurons. Human fetal astrocytes were infected with HIV1-CMV-GAD65-GFP (**Fig. 2K**) or HIV1-CMV-GFP (control) lentivirus and cultured for additional 7 days. After 7 days the culture media was replaced with fresh HEPES-buffered Tyrode's solution, incubated for 24 h and conditioned media (ACM) harvested. ACM was then applied into bath of whole-patch-clamped human NT neurons for 60 seconds. As shown by the trace of **Fig. 2 L** the application of ACM (measured GABA concentration: 8–14 µmol) induced inward current i.e. response consistent with GABA_A_ receptor-mediated depolarization [22], [23]. This response was similar to that measured after application of 50 µM GABA (**Fig. 2M**). Application of ACM harvested from astrocyte previously infected with control HIV1-CMV-GFP lentivirus was without response (**Fig. 2N**).

### Intrathecal delivery of GABA combined with systemic tiagabine treatment leads to potent anti-spasticity effect

The above *in vitro* data suggest that the use of HIV1-CMV-GAD65 lentivirus should similarly be effective in increasing GABA synthesis after targeted spinal parenchymal delivery and that such a local upregulation in GABA synthesis can lead to an anti-spasticity effect. Similarly, topical-intrathecal delivery of GABA should lead to a similar anti-spasticity effect.

**Figure 3:**
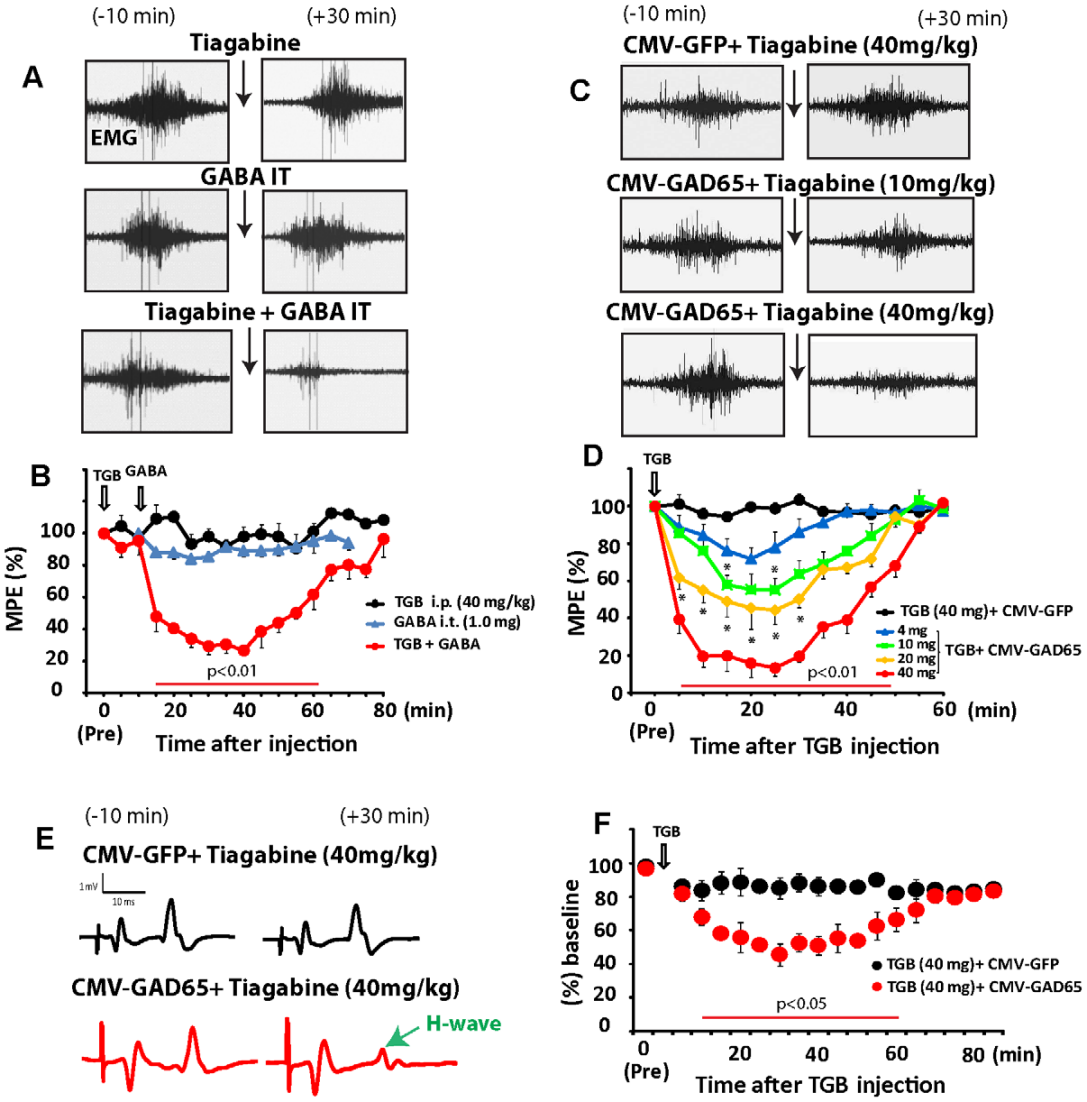
Effective suppression of spasticity after combined therapy with systemic tiagabine and intrathecal injection of GABA or spinal parenchymal GAD65 gene delivery. (**A**) EMG responses recorded from gastrocnemius muscle in spastic animals during computer-controlled ankle dorsiflexion before and after systemic treatment with tiagabine (40 mg/kg; i.p.; n = 6), intrathecal GABA (1 mg; IT; n = 6) or combined treatment with tiagabine+IT GABA (n = 6). (**B**) Time-course of ankle resistance measured during ankle dorsiflexion at baseline and then in 5-min intervals up to 80 min after treatments (* P<0.01; one-way analysis of variance-ANOVA, Bonferroni's posthoc test; MPE-maximum positive effect). (**C**) EMG responses recorded from the gastrocnemius muscle in spastic animals previously injected spinally with HIV1-CMV-GFP (control; n = 6) or HIV1-CMV-GAD65 (n = 6) lentivirus and then treated with systemic 10 mg/kg or 40 mg/kg tiagabine. (**D**) Time-course of anti-spastic effect after tiagabine treatment expressed as % of maximum possible effect in measured ankle resistance in HIV1-CMV-GFP or HIV1-CMV-GAD65-GFP lentivirus-injected animals (* P<0.01; one-way analysis of variance-ANOVA, Bonferroni's posthoc test; MPE-maximum positive effect). (**E**) Changes in H-wave amplitudes recorded from interdigital muscles of the lower extremity during high frequency (20 Hz) sciatic nerve stimulation in animals previously injected spinally with HIV1-CMV-GFP or HIV1-CMV-GAD65 lentivirus and then treated with 40 mg/kg tiagabine. (**F**) Time-course of changes in H-wave amplitudes before and up to 90 min after tiagabine administration (red line-P<0.05; unpaired *t* test).

To test this hypothesis we first injected GABA (1 mg) intrathecally in rats with developed ischemic spasticity and the effect on spasticity response measured during computer-controlled ankle dorsiflexion for 60 min. No significant anti-spasticity effect was seen (**Fig. 3A, B**). Because the baseline spinal extracellular concentration of GABA is in pmol range (20–30 pmol) [24], but µmolar (1–2 µmol) concentrations are required to exert its receptor-mediated effect on *in vitro* cultured neurons [25] we hypothesized that the activity of spinal GABA reuptake systems [26], [27] is likely responsible for the lack of sufficient GABA accumulation in the synaptic cleft after even high dose GABA administration. To address this we next tested the effect of systemic treatment with the GABA uptake inhibitor tiagabine, administered as a monotherapy or in combination with IT GABA. Systemic treatment with 40 mg/kg/i.p. tiagabine alone was without anti-spasticity effect (**Fig. 3A, B**). Because tiagabine has good permeability across the blood brain barrier and increases brain levels of extracellular GABA (2–3 fold increase) after systemic administration of 11.5–21 mg/kg of tiagabine [19] we speculate that the lack of functional effect in our model likely reflects the documented loss of GABA-producing interneurons in lumbar ischemia-injured segments. To validate this we next tested the effect of combined systemic treatment with tiagabine (40 mg/kg/i.p.) followed 15 min later by intrathecal injection of GABA (1 mg). In this combined therapy a potent and highly significant anti-spasticity effect was seen (**Fig. 3A, B**) the potency of which was similar to our previously reported anti-spasticity effect after intrathecal baclofen (GABA_B_ receptor agonist; 1 µg) treatment using the same spinal ischemia-induced spasticity model [21]. No suppressive effect on upper extremity motor function was noted and all animals showed continuing ability to move their upper limbs and grab food pellets if offered.

### Increase in spinal parenchymal GAD65 expression provides a potent antispasticity effect if combined with systemic tiagabine treatment

We next tested if spinal GAD65 overexpression will lead to increased local GABA release and if such a release will have a similar anti-spastic effect once combined with systemic tiagabine (1, 4, 10, 20 or 40 mg/kg) treatment. Spastic animals received a total of 20 bilateral injections of HIV1-CMV-GAD65-GFP (n = 6) or HIV1-CMV-GFP (n = 6; control) lentivirus targeted into ischemia-injured L2–L5 spinal segments and underwent spasticity assessments 7–21 days after virus delivery. In control HIV1-CMV-GFP-injected spastic animals, systemic administration of tiagabine (40 mg/kg,i.p.) was without effect (**Fig. 3C, D**). In contrast, in HIV1-CMV-GAD65-GFP-injected rats, treatment with tiagabine led to a potent and significant anti-spasticity effect. The peak effect was seen at 25 min after tiagabine administration and returned back to baseline by 60 min (**Fig. 3C, D**; p<0.01). Dose response analysis for tiagabine showed that doses ≥4 mg/kg provided significant (p<0.01) anti-spasticity effect at 15–25 min after tiagabine injection. No detectable effect on upper limb motor function was seen after tiagabine treatment and all animals showed continuing ability to move their upper limbs and grab food pellets if offered.

In separate experimental sessions, changes in H-reflex amplitudes evoked by high frequency stimulation was tested in ketamine-sedated animals. In spastic animals previously injected spinally with control lentivirus (HIV1-CMV-GFP; n = 6) no change in H-reflex amplitudes were seen up to 90 min after tiagabine injections (**Fig. 3E, F**). In animals receiving spinal injections of HIV1-CMV-GAD65-GFP lentivirus (n = 6) a significant (p<0.05) reduction of the H-wave amplitude was measured between 20–45 min after tiagabine injection and returned back to baseline by 65 min (**Fig. 3E, F**). Similar significant suppression of H-reflex activity in spastic patients after intrathecal baclofen treatment was reported [28], [29].

### Spinal parenchymal injection of HIV1-CMV-GAD65 lentivirus leads to a significant increase in GAD65 expression and extracellular GABA release in rat and minipig

**Figure 4:**
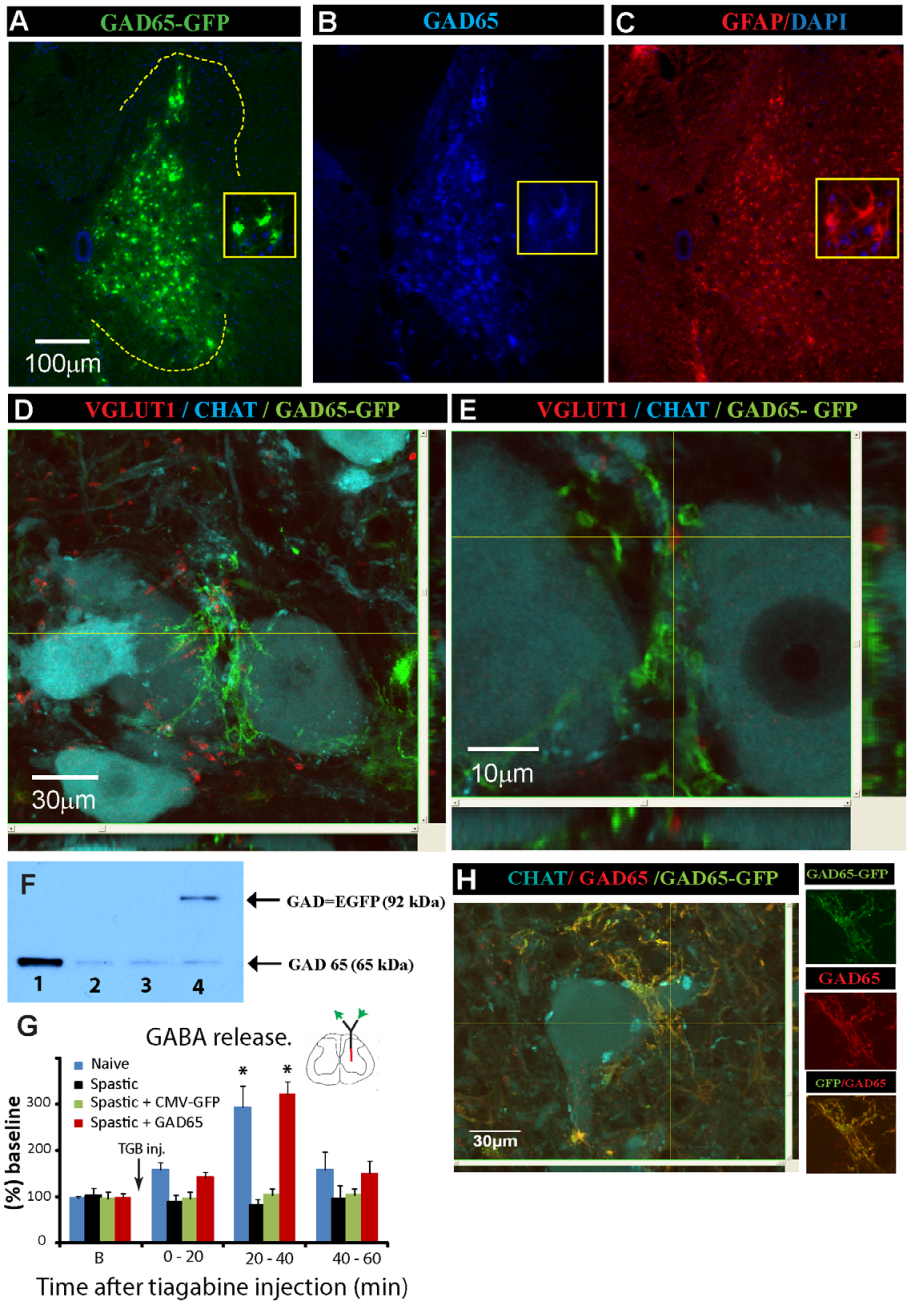
Spinal parenchymal injections of HIV1-CMV-GAD65-GFP lentivirus leads to increased GAD65 expression in infected astrocytes in rat and minipig and is associated with increased extracellular GABA release after tiagabine treatment in rats with ischemic spasticity. (**A–C**) Immunofluorescence images taken from a transverse lumbar spinal cord section of a spastic rat at 3 weeks after spinal injection of HIV1-CMV-GAD65-GFP lentivirus. Sections were stained with GFP, GAD65 and GFAP antibody. (**D, E**) Confocal images demonstrating the localization of GAD65-GFP (green) expressing processes in HIV1-CMV-GAD65-GFP-infected cells surrounding VGLUT1 (red)-IR primary afferent terminals in the vicinity of persisting CHAT (blue)-IR α-motoneurons. (**F**) Western blot analysis for GAD65 in spinal cord homogenate taken from lumbar spinal parenchyma of naive-control (column 1) spastic non-treated (columns 2 and 3) and spastic HIV1-CMV-GAD65-GFP-injected animal (column 4). (**G**) Extracellular GABA concentration measured by intraparenchymal microdialysis in lumbar gray matter in naive (n = 6), ischemic-spastic (n = 6), ischemic-spastic-HIV1-CMV-GFP (n = 6) and ischemic-spastic-HIV1-CMV-GAD65-GFP (n = 6) lentivirus-injected animals before and after systemic tiagabine (40 mg/kg) injection. A significant increase in extracellular GABA concentration was measured at 20–40 min after tiagabine administration in naive animals and ischemic-spastic animals previously injected spinally with HIV1-CMV-GAD65-GFP lentivirus (P<0.05; paired *t* test). (**H**) Confocal images of transverse spinal cord section taken from a minipig lumbar spinal cord at 2 months after spinal HIV1-CMV-GAD65-GFP injections and stained with GFP, GAD65 and CHAT antibody.

To identify the spinal laminar distribution and cellular specificity of HIV1-CMV-GAD65-GFP lentivirus-infected cells and to validate if such overexpression could increase spinal parenchymal GABA release, spastic rats received 20 bilateral injections (0.5 µl each) of HIV1-CMV-GAD65-GFP (n = 9) or HIV1-CMV-GFP (n = 9; control) lentivirus. At 14 days after lentivirus injection, GABA concentrations were measured in LVs-injected spinal segments using concentric microdialysis and HPLC. The presence of GAD65-GFP expressing cells was validated with immunofluorescence staining and quantified with western blotting. Histological analysis showed a preferential expression of the GAD65-GFP fusion gene in astrocytes (**Fig. 4A–C**). Numerous GFP+/GAD65+ astrocytic processes were identified in the vicinity of VGluT1-stained primary afferents residing next to the membranes of persisting CHAT-IR α-motoneurons (**Fig. 4D, E**). Western blot analyses of spinal cord homogenates prepared from lumbar spinal cord of naive, spastic non-treated, and spastic HIV1-CMV-GAD65-GFP-injected rats showed significant loss of GAD65 expression in spastic non-treated animals (if compared to naive control: see Fig. 1G) and the presence of the GAD65-GFP fusion protein in HIV1-CMV-GAD65-GFP-injected rats (**Fig. 4F**). Measurement of spinal extracellular GABA concentration before and after tiagabine (40 mg/kg; i.p.) administration showed a significant (p<0.05) increase in naïve and HIV1-CMV-GAD65-GFP lentivirus-injected spastic animals if compared to spastic (non-injected) or spastic HIV1-CMV-GFP lentivirus injected animals (**Fig. 4G**).

We next tested if spinal parenchymal injections of HIV1-CMV-GAD65-GFP in a preclinical minipig model (naive non-injured animals) would lead to a similar astrocyte-specific GAD65 upregulation. Guttingen-Minessota minipigs (n = 2) received 20 bilateral injections of LVs (6 µl each injection; 10 M.O.I) and survived for 1 or 2 months. Histological analysis of spinal cord sections at 1 or 2 months after LVs injection showed similar preferential astrocytic GFP-GAD65 co-expression in LVs injected spinal cord segments (**Fig. 4H**).

## Discussion

Decreased or completely lost activity of a facilitatory supraspinal input into spinal GABA-ergic inhibitory interneurons and resulting decrease in local segmental inhibition has been postulated as one of the key mechanisms leading to the development of muscle spasticity in patients with SCI [1], [2]. Comparably, loss of spinal inhibitory interneurons, as seen after transient episodes of spinal cord ischemia leads to development of functionally defined muscle spasticity and rigidity [21], [30]. Independent of the insult nature (e.g. spinal trauma or ischemia), clinical and experimental animal pharmacology studies have shown a comparable and potent antispasticity effect after systemic or spinal treatment with most commonly used antispasticity agent baclofen (GABA_B_ receptor agonist) [31], [32]. The primary site of baclofen-mediated hyperpolarizing action is believed to be at presynaptic Ia afferents [33], [34].

One of the major limitations of systemic baclofen treatment, however, is the lack of a localized spinal segment-restricted effect and relatively high doses required to achieve clinically relevant relief of spasticity frequently produce unwanted systemic side effects such as sedation [35]. Direct spinal delivery of baclofen using chronic intrathecal catheter provides a more site-restricted effect with less pronounced systemic acitivity, however it requires surgical intervention and ensuing complications associated with chronic intrathecal catheterization such as cerebrospinal fluid leak or infection has been described [32]. More importantly, limits of effective long-term use of IT baclofen include the development of baclofen tolerance (i.e. progressive escalation of dose to achieve consistent anti-spasticity effect) and withdrawal after an abrupt termination of baclofen treatment [36], [37].

Our study shows that animals with chronic ischemia-induced spasticity have a significant reduction in spinal parenchymal GAD65 expression which corresponds with a loss of GABA-ergic interneurons and GABA+ terminals on α-motoneuronal membranes and VGLUT1+ primary afferents. These data are in line with the postulated role of decreased GABA-ergic activity in the development of spinal ischemic spasticity. Spinal injection of lentivirus encoding the GAD65 gene targeted into ischemia-injured segments led to a significant increase in GAD65 expression primarily in astrocytes and was associated with increased extracellular GABA release once combined with systemic tiagabine treatment.

Preferential expression of GAD65 gene in infected astrocytes (as opposed to neurons) appears to provide a specific advantage with respect to expected GABA mediated anti-spasticity effect. As we have shown *in vitro*, infection of primary astrocytes led to a Ca2+ independent increase in extracellular GABA concentration. Accordingly, it is expected that astrocyte-mediated GABA release in the spinal parenchyma will be independent of the functionality and connectivity of local neuronal inhibitory circuitry and will specifically exert its hyperpolarizing effect on GABA_B_ receptor expressed on Ia afferents and/or α-motoneurons. The biological activity of astrocyte-produced GABA was confirmed by its depolarization-inducing effect on preferentially GABA_A_ receptor-expressing cultured hNT neurons (see Fig. 2).

Interestingly, the upregulation of spinal GAD65 expression in the absence of any other treatment, however, had no detectable anti-spastic effect. Previous studies have demonstrated that GABA concentrations required for an effective GABA_B_ receptor activation is in the µmol range [25]. We speculate that while a significant increase in GAD65 gene expression was achieved in lentivirus-infected regions, efficient GABA metabolism mediated in-part by the GABA reuptake system [26], [27] prevented effective GABA accumulation in the synaptic cleft and resulted in lack of any functional effect. In contrast, animals that had received lumbar injections of GAD65 lentivirus and were treated systemically with tiagabine (a GABA uptake inhibitor) exhibited a potent, dose-dependent reduction in spasticity of the lower extremities up to 60 min after tiagabine administration. Importantly, no detectable effect on the motor performance of the upper extremities (i.e. mediated by the activity of muscle groups innervated by virus non-injected cervical segments) was seen. In animals receiving lumbar injection of control GFP-tagged lentivirus no antispasicity effect was seen after the treatment with the same dose of tiagabine. Jointly these data show that the use of tiagabine at doses which have no significant therapeutic anti-spatic effect nor detectable side effects when used as a monotherapy is highly effective in increasing local GABA-ergic inhibitory tone in GAD65-overexpressing spinal cord regions; the magnitude of such increased local inhibition provides a clinically-relevant relief of spasticity.

We believe, that the ability of such combined therapy in which systemically administered drugs (such as tiagabine) is effective in regulating the activity of the therapeutic product (GABA) in remote GAD65 gene-overexpressig sites can potentially have a significant clinical implications.

**First**, the identity of specific spinal segments innervating the affected spastic muscle groups can be neurologically mapped, lateralized and selected for the segment/site-specific GAD65 gene delivery.

**Second**, extensive clinical data show a potent anti-spastic effect after intrathecal baclofen delivery and this effect is independent on the spinal or supraspinal origin of spasticity [17]. Thus, it is likely that spinal segmental GAD65 upregulation once combined with systemic GABA uptake inhibitor treatment will have a similar therapeutic effect in spasticity of supraspinal and spinal origin.

**Third**, comparable site-specific delivery of GAD65-encoding vectors targeting functionally/electrophysiologically-defined brain epileptic foci can be performed. Previous data from other laboratories have confirmed an improvement in the parkinsonian behavioural phenotype and neuronal rescue after AAV-CBA-GAD65 delivery into the subthalamic nucleus in 6-OHDA–lesioned rats [38]. We speculate that proposed combination treatments can lead to a more pronounced anti-epileptic effect with less side effects such as general sedation.

**Fourth**, the serum half-life of tiagabine in human patients is between 5–8 hrs (in contrast to 55 min in rats) and therefore comparable duration of the antispasticty effect can be expected in human patients once combined with spinal parenchymal GAD65 gene delivery [39], [40], [41].

Our current study employed a CMV-promoter-driven lentiviral construct encoding GAD65 and astrocytes were the primary cells expressing the GAD65-GFP transgene both *in vitro* and in vivo. In addition to the rat spasticity model, testing the same lentivirus in a preclinical non-injured minipig model showed a similar expression profile and a stable expression of GAD65-GFP protein in astrocytes at 1 and 2 months after spinal lentivirus injections. This is consistent with recent studies that showed preferential astrocytic expression of GFP in spinal gray matter after direct parenchymal delivery of HIV1-CMV-EGFP lentivirus in rat [42].

In addition to cell integrating gene transfer after the use lentiviral vectors, there are reports of successful GAD65 gene overexpression after AAV-GAD65 injections into subthalamic nuclei. In those studies, persistent GAD65 expression was seen up to 4–5 months after AAV-GAD65 injections [38]. More importantly, recent systematic data demonstrate a high efficiency of AAV-based gene delivery into rat or minipig striatum even after a limited number of AAV injections (1–2 injections) [43], [44], [45], [46]. Thus the use of AAV-based, genome-non-integrating GAD65-encoding vectors appears to have the most favourable profile to be used in clinical settings with fewer injections required to achieve segment-specific GAD65 expression.

In summary we demonstrate that the treatment with the orally bioavailable GABA-mimetic drug tiagabine if combined with spinal-segment specific GAD65 overexpression is highly effective in suppressing chronic muscle spasticity. This combined treatment can represent a novel therapeutic approach to modulate chronic spasticity in patients after spinal traumatic or ischemic injury.

## Methods

### Ethics Statement and Institutional Animal Care and Use Committee approvals

This study was approved by the University of California, San Diego (UCSD) Internal Review Board (IRB), approval ID#101323.

All animal studies were carried out under protocols approved by the Institutional Animal Care and Use Committee at University of California (approval Ids # S01193 and S07016) San Diego or of the Czech Academy of Sciences (IAPG Libechov: approval ID# 5/2010) and were in compliance with *The Association for Assessment of Laboratory Animal Care* guidelines for animal use. All studies were performed in such a manner as to minimize group size and animal suffering.

### Induction of spinal ischemic spasticity in rat

Transient spinal cord ischemia (10 min) was induced as previously described [47]. Briefly, in isoflurane (1.5–2%)-anesthetized SD rats, a 2F Fogarty catheter (Am.V. Muller, CV 1035; Baxter, Inc., Irvine, CA, USA) was passed through the left femoral artery to the descending thoracic aorta to the level of the left subclavian artery. Distal arterial pressure (i.e. below the level of aortic occlusion) was monitored by cannulation of the tail artery with PE-50 catheter. Spinal cord ischemia was induced by inflation of the intra-aortic balloon catheter (0.05 ml of saline) and concurrent systemic hypotension (40 mm Hg) induced by blood withdrawal (10.5–11 cc into a heated (37°C) external reservoir) via a 20- gauge polytetrafluoroethylene catheter placed in the left carotid artery. The efficacy of the occlusion was demonstrated by an immediate and sustained drop in distal blood pressure. After 10-min ischemia, the balloon was deflated, and the blood was reinfused. When the arterial blood pressure was stabilized (20–30 min after reflow), the arterial lines were removed, wounds closed and animals were allowed to recover. In this spinal ischemic model on average 50–60% of animals exposed to 10 min of aortic occlusion show development of progressive muscle spasticity at 5–21 days after ischemia.

### Identification of muscle spasticity in rats with spinal ischemic injury

One to eight weeks after ischemia, animals were tested for the presence of spasticity. Spasticity was identified as an increase in ankle resistance during computer-controlled ankle dorsiflexion, which correlated with increased EMG activity measured in the gastrocnemius muscle during the same time frame. Direct measurement of ankle resistance during computer-controlled ankle dorsiflexion was performed as described previously [5]. Rats were individually placed in a plastic restrainer, and one hind paw was securely fastened to the paw attachment metal plate, which is interconnected loosely to the “bridging” force transducer (LCL454G, 0–454 g range; or LCL816G, 0–816 g range; Omega, Stamford, CT). After a 20 min acclimation period, rotational force was applied to the paw attachment unit using a computer-controlled stepping motor (MDrive 34 with onboard electronics; microstep resolution to 256 microsteps/full step; Intelligent Motion Systems, Marlborough, CT), causing the ankle to dorsiflex (Fig. 1O). The resistance of the ankle was measured during 45° of dorsiflexion lasting 3 sec (15° s^−1^), and data were collected directly to a computer using custom software (Spasticity version 2.01; Ellipse, Kosice, Slovak Republic).

To identify the mechanical component of measured ankle resistance, all animals were anesthetized with 2.5–3% isoflurane at the end of the experiment and the relative contribution of mechanical vs. neurogenic component (isoflurane-sensitive) was calculated. Data generated before and after treatment were expressed as % of maximum possible effect of neurogenic component contributing to measured resistance. Each recorded value was the average of three repetitions. To record EMG activity, a pair of tungsten electrodes was inserted percutaneously into the gastrocnemius muscle 1 cm apart. EMG signals were bandpass filtered (100 Hz to 10 kHz) and recorded before, during, and after ankle dorsiflexion. EMG responses were recorded with an alternating current-coupled differential amplifier (model DB4; World Precision Instruments, Sarasota, FL) and stored on a computer for subsequent analysis. EMG was recorded concurrently with ankle resistance measurement during dorsiflexion.

### Intrathecal catheterization

In some animals intrathecal catheters were implanted into lumbar intrathecal space [48]. Under isoflurane anaesthesia, an 8.5 cm PE-5 catheter (Spectranetics, Colorado Springs, CO) connected to 4 cm of PE-10 was inserted into the intrathecal space through an incision in the atlanto-occipital membrane of the cisterna magna. The PE-10 arm was externalized on the neck for bolus drug (GABA) delivery or for colchicine injections.

### Construction and preparation of lentivirus vectors

Rat GAD65 cDNA, inserted into the EcoRI site of the pBluescript-SK (Stratagene, CA), was obtained from Dr. A. Tobin (UCLA) [49]. HIV1 vector backbone plasmid pHIV7 containing the WPRE and cPPT sequences were obtained from Dr. J-K. Yee (City of Hope) [50]. To construct the HIV1 vector expressing the GAD65 cDNA from hCMV promoter, hCMV promoter was isolated from pLenti6/V5-GW/lacZ (Invitrogen, CA) with ClaI-EcoRV digestion and inserted into the ClaI-EcoRV sites of the pBluescript-GAD65. The hCMV-GAD65 cassette was then isolated and inserted into the Bam HI site of the pHIV7 and the resulting plasmid was designated pHIV7-CMV-GAD65. Similarly, to construct the HIV1 vector expressing GAD65-EGFP fusion gene, the GAD65 cDNA was inserted downstream of the hCMV promoter of the pEGFP-N1 (Clontech, CA) adjusting the reading frame with the downstream EGFP gene. The hCMV-GAD65-EGFP cassette was isolated and then inserted into the BamHI site of the pHIV7 to create the HIV1 vector pHIV7-CMV-GAD65-EGFP. A control HIV1 vector pHIV7-CMV-EGFP expressing EGFP gene from the same hCMV promoter was constructed by inserting the hCMV-EGFP unit isolated from the pEGFP-N1 into the pHIV7.

Lentivirus vectors were produced by transient co-transfection of HEK293T cells (Invitrogen, CA; Cat.No: R70007) maintained in Dulbecco's modified Eagle's medium (DMEM) with 10% FCS. 293T cells in 150 mm dishes were co-transfected by the CaPO4-DNA co-precipitation method with each HIV1 vector plasmid, pLP1 and pLP2 (Invitrogen, CA), and pCMV-G [51]. Conditioned media at day 1, 2 and 3 post transfection were collected, filtered through a 0.45 µm filter, and concentrated by centrifugation at 7000 rpm for 16 hrs at 4°C with a Sorvall GS-3 rotor. The resulting pellets were resuspended with buffer containing 10 mM Tris HCl, pH 7.8, 1 mM MgCl_2_ and 3% sucrose.

Infectious titters of the HIV1 vectors were measured by real-time Q-PCR using the HIV1-CMV-GFP vector (1×10^−9^ iu/ml) as the standard. HEK293T cells in a 6-well plate were infected with serially diluted vector preparations in the presence of polybrene (4 µg/ml). Infected cells were passaged once every 4 days and cell DNAs were prepared at day 14 post infection by the DNeasy Blood & Tissue kit (Qiagen Science, MD). Real-time Q-PCR was performed to measure the copy numbers of the provirus in the chromosome of the infected cells using a primer set selected from the WPRE sequence and the final virus titters were adjusted to 1×10^−9^ iu/ml.

### Lentiviral infection of primary spinal cord cultures

Spinal cords were isolated from embryonic day 14 (E14) Sprague–Dawley rats (Harlan Sprague–Dawley Inc., Indianapolis, IN). Cells were isolated using the papain dissociation system (Worthington Biochemical Corp., Freehold, NJ), following the manufacturer's instructions with modification. Tissue was dissociated in 5 ml of papain dissociation solution by trituration, followed by agitation for 20 min at 37°C in 5% CO_2_. The cell suspension was centrifuged at 300× *g* for 5 min and the cell pellet was resuspended in Deact solution (albumin-ovomucoid inhibitor/DNase solution). Resuspended cells were then layered on top of a discontinuous density gradient of albumin-ovomucoid inhibitor mixture and then centrifuged at 70× *g* for 5 min. The cell pellet was resuspended in 50 µl of 10 M.O.I. HIV1-CMV-GFP, HIV1-CMV-GAD65 or HIV1-CMV-GAD65-GFP lentivirus and incubated at 37°C for 10 min. Infected cells were then plated into poly-d-lysine-coated chamber slides and cultured for 1–3 weeks in growth medium (DMEM high glucose supplemented with 10% FBS, 2 mM l-glutamine, B27 supplement and 100 U/ml penicillin and 100 µg/ml streptomycin; GIBCO, Grand Island, NY). At 1–3 weeks some cells were washed with PBS 3× and then fixed with 4% paraformaldehyde for 30 min at RT and later used for immunofluorescence staining.

### Measurement of extracellular GABA release in rat primary spinal cord culture and human fetal astrocyte culture

Tissue culture media was collected from cultured rat spinal cord cells at baseline and then at 3 days, 7 days and 2 weeks after infection with HIV1-CMV-GFP, HIV1-CMV-GAD65 or HIV1-CMV-GAD65-GFP lentivirus and filtered through 0.22 µm filter. At 14 days cultures were washed with PBS solution 3× and incubated in Ca^2+^ free PBS for 3 hrs. Samples were collected at baseline and then at 1, 2 and 3 hrs. All samples were analyzed for GABA concentration using HPLC (HTEC-500; EICOM, Japan).

Human fetal astrocytes (ScienCell, Carlsbad, CA, USA) were infected with HIV1-CMV-GAD65-GFP or HIV1-CMV-GFP (control) lentivirus and cultured for additional 7 days in DMEM/F12+10%FBS. After 7 days the culture media was replaced with fresh HEPES-buffered Tyrode's solution, incubated for 24 h and conditioned media (ACM) harvested for GABA measurement and for patch clamp experiment using cultured human NT neurons (see following paragraph).

### Patch clamp recordings

Human NT neurons (Layton Biosciences) [52] were co-cultured with human fetal astrocytes (ScienCell, Carlsbad, CA, USA) using DMEM/F12 +10% FBS for 3–4 weeks. The recording micropipettes (tip resistance 4–6 mOm) were filled with internal solution: 135 mM K-gluconate, 4 mM MgCl_2_, 10 mM HEPES, 10 mM EGTA, 4 mM Mg-ATP and 0.2 mM Na-GTP [pH 7.4]. Recordings were made using a MultiClamp 700B amplifier and Digidata 1440A interface (Molecular Devices). Signals were filtered at 10 kHz and sampled at 10 kHz. The whole-cell capacitance was fully compensated. The bath was constantly perfused with fresh HEPES-buffered saline: 140 mM NaCl, 5 mM KCl, 10 mM HEPES, 1 mM EGTA, 3 mM MgCl_2_, 10 mM glucose [pH 7.4]. Cells were visualized using an OLYMPUS BX51W1 fixed-stage upright microscope. Whole-cell recordings were carried out at a holding potential of −60 mV in gap-free mode. Cell were constantly perfused with extracellular solution with flow rate 2 ml/min. Astrocyte-conditioned media (ACM) or 50 µmol GABA (Sigma) was applied to the bath solution when the holding current was stable for at least 4 min. All recordings were performed at room temperature.

### Spinal parenchymal lentivirus injection in spastic rat and naive minipig

Rats with identified spasticity were anesthetized with 1.5–2% isoflurane (in room air), placed into a spinal unit apparatus (Stoelting, Wood Dale, IL, USA) and a partial Th12–L1 laminectomy was performed using a dental drill (exposing the dorsal surface of L2–L6 segments). Using a glass capillary (tip diameter 80–100 µm) connected to a pressure-controlled microinjector (Stoelting), rats were injected with 0.5 µl of the HIV1-CMV-GAD65-GFP (n = 12) or HIV1-CMV-GFP (control; n = 12) lentivirus (10 M.O.I.). Animals received a total of 10 bilateral injections. The duration of each injection was 60 s followed by 30-s pause before capillary withdrawal. The injection was targeted into central gray matter (laminae V–VII) (distance from the dorsal surface of the spinal cord at L3 level: 1 mm) [53]. The rostrocaudal distance between individual injections ranged between 1000–1500 µm. After virus injections, the incision was cleaned with 3% H_2_O_2_ and penicillin/streptomycin mixture and closed in two layers. After LVs injections animals were allowed to recover for minimum of 10 days before the effect of LVs injections on the magnitude of spasticity was measured.

Minnesota-Gottingen minipigs (males; 18–23 kg; n = 2) were premedicated with intramuscular azaperone (2 mg/kg ; Biotika, SK) and atropine (1 mg/kg; Biotika, SK) and then induced with ketamine (20 mg/kg, i.v.). After induction, animals were intubated with a 2.5F tracheal tube. Anesthesia was maintained with a 1.5% isoflurane in 50/50% air -oxygen mixture at a constant 2 L/min flow rate. Oxygen saturation was monitored throughout the procedure using a pulse oximeter (Nellcor Puritan Bennett Inc., Ireland). To immobilize the lumbar spinal cord animals were mounted into a spinal immobilization apparatus and the lumbar portion of the animal was lifted 5″ above the operating table to eliminate spinal cord pulsation due to respiration [54]. A dorsal laminectomy of L2–L5 vertebrae, corresponding to L3–L6 spinal segments in minipigs, was then performed and epidural fat removed using cotton swabs. The dura was left intact. To deliver LVs, an XYZ manipulator (M325; WPI, Sarasota, FL, USA) was used and mounted directly to the operating table. A Hamilton syringe with a 30 gauge needle was then mounted into the manipulator and connected to a microinjector (Stoelting) using PE-50 tubing. To connect the PE-50 tubing to the Hamilton syringe the plunger was removed and one end of the PE-50 tubing was inserted 1 cm into the syringe and sealed with silicone. Animals then received a total of 20 injections (10 on each side) of HIV1-CMV-GAD65-GFP lentivirus (10 M.O.I.; 6 µl each) targeted into intermediate zone (lamina VII) of L2–L4 segments (distance from the dorsal surface of the spinal cord at L3 level: 3–3.5 mm). The distance between individual injections was 1–1.5 mm. All surgical interventions followed rigid aseptic procedures. All materials were subjected to autoclaving or gas sterilization. After LVs injections animals survived for 1 or 2 months.

### Hoffmann reflex recording

H-reflex was recorded as previously described [21]. Under ketamine anesthesia (100 mg/kg/hr, i.m.) the right hind limb of the animal was secured and a pair of stimulating needle electrodes was transcutaneously inserted into the surroundings of the tibial nerve. For recording a pair of silver needle electrodes was placed into the interosseous muscles between the fourth and the fifth or the first and the second metatarsal right foot muscles. The tibial nerve was stimulated using square pulses with increasing stimulus intensity (0.1–10 mA in 0.5 mA increments, 0.1 Hz, 0.2 ms; WPI; Isostim A320) and responses were recorded with an A/C-coupled differential amplifier (Model DB4; DPI, Sarasota, FL). After the M-max and H-max responses were identified the intensity of stimulus which evoked H-max amplitudes were used in subsequent high frequency (20 Hz) stimulation experiment. In HIV1-CMV-GAD65-GFP (n = 6) or HIV1-CMV-GFP (n = 6) lentivirus-injected animals a high frequency stimulation was performed in 5 min intervals for up to 90 min after tiagabine (40 mg/kg, i.p.) injection. Changes in H-wave amplitude were then compared between both lentivirus-injected groups.

### Spinal cord microdialysis and extracellular GABA release measurement

Spinal cord microdialysis was performed in naive (n = 6), spastic-non treated (n = 6), spastic HIV1-CMV-GFP lentivirus-injected (n = 6) and spastic HIV1-CMV-GAD65-GFP (n = 6) lentivirus-injected animals. Rats were anesthetized with 2% isoflurane, previous laminectomy site (in lentivirus injected animals) re-exposed and concentric microdialysis probe (A-2-8-02; cut off: MW 50, 000; EICOM, Japan) placed into central gray matter between L3–L6 spinal cord segments. Microdialysis fiber was perfused with artificial CSF at 2 µl/min and samples collected on dry ice. After 120 min washout samples were collected in 20 min intervals before and after tiagabine (40 mg/kg, i.p.) injections and analyzed for GABA using HPLC (HTEC-500; EICOM).

### Intrathecal administration of colchicine

To decrease axonal transport of GABA and increase its concentration in the neuronal soma, control animals (n = 3) and animals with ischemic spasticity (n = 3) previously implanted with PE-10 intrathecal catheters received intrathecal bolus injection of colchicine (10 µl, 1% in saline) 48 h prior to sacrifice. This treatment was used to determine the presence or loss of GABA-ergic neurons in animals with ischemic spasticity. These animals were not used in any functional measurements.

### In vivo perfusion fixation and tissue processing

At the end of the survival periods, animals were anesthetized with pentobarbital (40 mg/kg; i.p.) and transcardially perfused with heparinized saline (100 ml-rat; 5l-minipig) followed by 4% paraformaldehyde in 0.1 M phosphate buffer (PB; 500 ml-rat; 5l-minipig). The spinal cords were dissected and postfixed in the same fixative overnight at 4°C. After postfixation tissue was cryoprotected in graded sucrose solutions (10, 20 and 30%). For GABA staining animals were perfused with 2% paraformaldehyde +0.3% glutaraldehyde solution.

### Immunofluorescence staining

A standard immunofluoresence staining protocol was followed. After cryoprotection frozen coronal spinal cord sections (20–30 µm) were cut. Free floating sections were placed in PBS (0.1 M; pH = 7.4) containing 5% normal donkey serum (NDS), 0.2% Triton X-100 (TX), for 2 h at room temperature to block non-specific background. This was followed by overnight incubation at 4°C with the following primary antibodies: mouse anti- GAD65(1∶500; Developmental Studies Hybridoma Bank, University of Iowa, Iowa City, IA); rabbit anti-GAD65 (1∶1000, Chemicon), mouse anti-GAD67 (1∶1000, Chemicon), rabbit anti-GFAP (1∶500; Chemicon); mouse anti-VGLUT1-3 (1∶2000; Chemicon Inc.), goat anti-CHAT (1∶100; Chemicon Inc.), mouse anti-GABA (1∶15000; Chemicon), guinea pig-anti GABA B R1 and R2 (1∶2000; Millipore Inc.,), rabbit anti-synaptophysin (SYN) (1∶200; Novocastra Laboratory). After incubation with primary antibodies, sections were washed 3× in PBS and incubated with fluorescent-conjugated secondary donkey anti-rabbit, donkey anti-mouse, donkey anti-goat and donkey anti-guinea pig antibodies (Alexa 488, 594, 680; 4 µl/ml; Molecular Probes, Eugene, OR, USA). All blocking and antibody preparations were made in 0.1 M PBS/0.2% TX/ in 5% normal donkey serum. For general nuclear staining DAPI (1 µl/ml) was added to the final secondary antibody solutions. After staining, sections were mounted on slides, dried at room temperature and covered with Prolong anti-fade kit (Molecular Probes). Stained sections were analyzed and photographed with epifluorescence microscope (AX70; Olympus) and confocal microscope (Fluoview 1000, Olympus).

### Quantitative analysis of GABA neurons, GAD65/67 and VGLUT1 terminals and α-motoneuron-expressed GABA B R1+R2 receptor in L2–L5 spinal segments

#### GABA-ergic cell bodies

Five sections from each animal were stained for GABA and a blinded investigator counted all GABA-positive neuronal bodies using UTHSCA Imagetool (developed at the University of Texas Health Science Center at San Antonio, Texas, USA); the same limits for pixel intensity and structure size were set in all images analyzed.

#### VGluT1/GAD65/GAD67-positive terminals

Analysis was performed according to Todd et al. [55] and Hughes et al. [56]. Briefly, 3 sections were selected from each rat (3 naive and 3 ischemic) and analysed by confocal microscopy (Leica Microsystems, Bannockburn, IL) using a 100× oil-imersion objective with 2× zoom (200× magnification, 75×75 µm field size). In all cases sequential scanning with the 488, 543, and 647 nm laser lines was used to capture two random scan fields (8–10 optical layers, z-separation 0.5) in lamina IX with identical confocal settings for all images. Firstly, using images constructed from 2 optical layers, the total number of immunoreactive structures in each scan field was counted using UTHSCA Imagetool with the same limits for pixel intensity. Secondly, for each VGluT1 terminal in the constructed image, the number of GAD65 and GAD67 boutons in contact with each VGluT1-IR terminal was counted by a blinded investigator. Thirdly, each VGluT1-positive terminal identified in the constructed images was classified as having contact with zero (no inhibitory contact) or 1 or more GAD65/67 boutons (some inhibitory contact).

#### GABA-ergic input to motoneuron cell bodies

Three sections from each animal were stained for GABA, synaptophysin and ChAT and confocal microscope (Leica Microsystems, Bannockburn, IL) images of lamina IX α-motoneurons were captured using a 100× oil-immersion objective; the same settings were used to capture all images. GABA-IR terminals were identified and only those that were double-labelled with synaptophysin and in contact with the motor cell soma (not associated processes) were counted. A total of 89 or 58 cells were assessed from naive and ischemic-spastic animals, respectively.

#### GABA B R1+R2 receptor in lumbar α-motoneurons

Three sections were selected from each rat (3 naive and 3 spastic) and analysed using digital images captured with 20× objective (Leica BMX). The total number of GABA B R1 or R2 immunoreactive punctata in each NeuN+ α-motoneuron (cell body size <700 µm^2^) in the ventral horn was counted using UTHSCA Image tool with the same limits for pixel intensity. Population distribution of immunoreactive receptors (punctata) with different intensity was then calculated and used for statistical analysis.

### Western blot analysis of GAD65 and GAD67 protein in rat spinal cord

Samples of L2–L6 spinal cord segments were collected by hydroextrusion from naive (n = 3), spastic non-treated (n = 3) and spastic-HIV1-CMV-GAD65-GFP-injected animals (n = 3). Harvested segments were cryo-sectioned (40 µm thick sections) and lysed for 30 minutes using lysis buffer containing 50 mM Tris (pH 7.4) (5429.3; Roth), 250 mM NaCl (3957.2; Roth), 5 mM EDTA (E5134; Sigma), 50 mM NaF (S-1504; Sigma), 1 mM Na_3_VO_4_ (S6508; Sigma) in 1% Triton® X-100 (T8532; Sigma) with protease inhibitor cocktail tablets (Complete Mini, EDTA-free; 11836170001; Roche) and 1 mM phenylmethylsulphonyl fluoride (PMSF; 837091; Roche). All samples were sonicated in a cold water bath for 5 minutes, followed by centrifugation at 10000× g at 4°C for 20 min. Total protein levels were determined by the BCA Protein Assay method (23225, Thermo Scientific). Samples were incubated with sample buffer (distilled water with 125 mM Tris-HCl, 4% SDS, 20% glycerol, 10% 2-mercaptoethanol, 0.004% bromophenol blue) at 95°C for 5 minutes and 15 micrograms of total protein was loaded from each sample on the 10% gel acrylamide gel. After electrophoresis, proteins were transferred to nitrocellulose membrane (Trans-Blot, 0.45 micrometer, Bio-Rad, CA) using a semidry blotting system (TE70XP, Hoefer, USA). The membrane was blocked with 5% nonfat dry milk (NFDM) in Tris-buffered saline with 0.5% Tween 20 (TBS-T, pH 7.4), incubated with the primary antibody (mouse anti-GAD65 or-GAD67 (Hybridoma Bank, Iowa) diluted 1∶200 in 5% NFDM in TBS-T) overnight at 4°C on a shaker. The next day, the membrane was washed 3 times in TBS-T and consequently incubated in donkey anti-mouse Ab HRP conjugate, (Jackson Lab, full source info) diluted 1∶10000 in 5% NFDM in TBS-T for one hour at RT with gentle shaking. SuperSignal West Pico Chemiluminescent Substrate (34077, Pierce) detection system was used for visualization. Western blotting signal was quantified by determining the grey values of given band using the ImageJ software.

### Statistical analysis

Statistical analysis of spasticity data was performed with one way ANOVA followed by Bonferroni post hoc test. H-reflex data were analyzed using un-paired *t*-test. The GABA release data were analyzed using paired *t*-test. Statistical analysis of GABA-ergic neurons and GABA+; GAD65/67 terminals was performed using unpaired *t*-test. Values of P<0.05 were considered significant.
